# The Association between Screen Time and Weight Status in Hispanic Children

**Published:** 2015-09-04

**Authors:** M Doherty, M Santiago-Torres, Y Cui, D Schoeller, T LaRowe, A Adams, A Carrel

**Affiliations:** 1 Nutritional Sciences, University of Wisconsin-Madison, Madison, WI, USA; 2 Engineering, University of Wisconsin-Madison, Madison, WI, USA; 3 Department of Family Medicine, University of Wisconsin-Madison, Madison, WI, USA; 4 Department of Pediatrics, University of Wisconsin-Madison, Madison, WI, USA

## Abstract

**Background:**

About one-third of U.S. children are overweight or obese and the number is even higher among Hispanics children (41%). In this regards, the time spent in sedentary behaviours is higher among Hispanic children versus non-Hispanic white children. But whether the home environment contributes to the obesity disparity among Hispanic children through the promotion of sedentary behaviours at home is less known. We aimed to investigate the associations between the home environment, parental limiting, and screen time with Hispanic children’s body weight.

**Methods:**

Study participants were middle school Hispanic children (n=187), ages 10–14 years and their parents. Children’s anthropometrics were measured and used to calculate BMI z-scores. Questionnaires were used to assess children’s time spent on physical activity (PA), sedentary activities, and to query parents on the home environment and parental limiting.

**Results:**

Total time (h/d) spent watching television (TV) was positively associated with children’s BMI z-score (*P*=0.02). However, no association was found between total screen time (TV, video games, and computer) and PA and with children’s BMI z-score. Sleeping time (h/d) was inversely associated with children’s BMI z-score (P=0.02); while there was a significant interaction between sleeping time and gender (*P*-interaction=0.02). Further, having a screen in the bedroom was positively associated with children’s TV and total screen time (*P*<0.05); while parental limits on screen time was inversely associated with children’s screen time (*P*<0.05).

**Conclusions:**

Screen and sleep time may contribute to higher body weight among Hispanic children, independently of associations with physical activity. Our findings suggest a differential effect of gender in the contribution of sleep time to higher body weight, in that girls spent less time sleeping when compared to boys. These findings can inform obesity-prevention efforts to intervene at the family level in improving sleeping patterns and increasing physical activity while reducing sedentary opportunities at home.

## Introduction

Although annual increases have slowed, there remains an obesity epidemic in the United States (U.S.) such that over one-third of adults are obese [1]. Children have not been spared. About one-sixth of children are obese and one third of children aged 6 to 19 are either overweight or obese [2]. This number is even higher among Hispanic children, with about 41% of children in this age range considered overweight or obese [3]. Obesity at a young age puts individuals at greater risk for serious health problems, including hypertension, diabetes, high cholesterol, and cardiovascular disease as well as in adulthood [3].

The increase in the prevalence of excess weight in children is a complex interaction between genetics and environmental factors, including modifiable behaviours, such as diet, physical activity (PA) and sedentary behaviours [3]. Herein, we concentrate on the later of these modifiable risk factors. Coinciding with the obesity epidemic has been an increase in total screen time among U.S. children, which is considered a sedentary behaviour associated with technological advancements in the past fifty years giving children greater access to televisions, video games, and computers. Numerous studies have found a positive association between total screen time and body mass index (BMI) among children and adolescents in the U.S. [4–6]. The literature most strongly supports the correlation between watching television and BMI [7–13]. In contrast, the results for investigations of video gaming and non-homework computer usage have not reached a consensus on their relation to BMI [11, 13, 14]. Furthermore, having a television, video game system or computer (screen) in the bedroom has shown to be correlated to higher BMIs in children [4, 15, 16]; whereas parental limits on screen time have been shown to have a negative correlation with the time children spend on these activities [17]. Although, the American Academy of Paediatrics (AAP) recommends no more than two hours of screen time per day and the removal of televisions and other screens from children’s bedrooms as a way of possibly preventing excessive weight gain in children, many children do not meet these recommendations [3, 7, 18].

While there is a great deal of literature on the topic of sedentary behaviour and screen time’s association with BMI and its mediating factors in children, there is little that specifically focuses on Hispanic children, who are at a higher risk for obesity when compared to their non-Hispanic counterparts. It is also unclear whether sedentary behaviours at home and parental limitations of these activities contribute to Hispanic children’s body weight and therefore, risk of metabolic disease. To try and understand the underlying behaviours that may contribute to higher sedentary time in Hispanic children within the home environment, the present study aimed to investigate the associations between the home environment, parental limiting, screen, and sleeping time with the body weight of Hispanic children from an urban community in the U.S. Secondly, we aimed to evaluate specific factors at home that may mediate the association between screen time and Hispanic children’s body weight.

## Methods

### Study Population and Measurements

The present study used data collected for the HAPPY (Healthy Activities Partnership Program for Youth) project, a cross-sectional study among Hispanic children and their parents performed during the 2010–2011 school year. Study recruitment and enrolment has been previously reported [19, 20]. Briefly, the cohort consisted of 187 Hispanic students from the Bruce-Guadalupe Community School in Milwaukee, WI part of the United Community Centre (UCC). The students ranged in age from 10–14 years old (48% male). Students’ height was measured using a stadiometer and their weight using a beam balance scale during physical education class at the school, without shoes and in light clothing. Height and weight measures were used to calculate children’s BMI z-scores and BMI percentile categories for age and sex.

Responses from three questionnaires were used in the present study– two completed by the children, an activity diary and an activity survey, and one home environment questionnaire completed by the parents. The activity diary questionnaires a previous-day activity recall method to assess children’s daily activity patterns and participation previously tested for reliability by Cui et al, 2012 [21]. It included questions completed by the children about daily time (in 15 min intervals) children spent watching TV, using the computer, playing video game, using the phone and studying or reading for two week days and one weekend day. The children also reported the time they went to bed and woke up on the activity diary, which was used to calculate the average amount of sleep per day. In the activity survey, children also provided information on which, if any, of these activities their parents set time limits during weekdays, weekends or both. The final data was obtained from the home environment questionnaire, in which parents were queried whether a screen – defined as a TV, computer, and/or video game – was present in their child’s bedroom.

### Statistical Analysis

Sample characteristics were summarized by standard descriptive analysis in terms of mean ± standard deviation for continuous variables and as percentages for categorical variables. Pearson correlation coefficients were calculated to assess the relation between the categorical variable – TV in the bedroom – and the continuous variables. Children’s daily time spent watching TV was averaged and weighted accordingly from time spent on weekdays (2 days) and one weekend day as reported by the children. The same analysis was performed for video game time and non-homework computer usage. Following this, time spent watching TV, playing video games, and non-homework computer usage were summed to create the screen time variable. Given that the children attended the same school, they reported approximately the same amount of hours “studying or in class,” which averaged approximately 8 hours per day (h/d). We found no association between time spent in school and children’s body weight (data not shown). Children were asked to mark off which activities – TV watching, video gaming, and/or non-homework computer usage – were limited by their parents on weekdays or weekends. Negative answers (no vs. yes) were given a value of zero and positive answers (yes vs. no) were given a value of one. Parental limiting for each of the sedentary activities were weighted with 5/7 given to weekday values and 2/7 given to weekend values to calculate weekly distribution on a scale of zero to one.

General linear regression models were used to investigate the associations between home environment characteristics, parental limiting, time spent in sedentary activities, and sleeping time with the body weight of Hispanic children. All linear regression models were adjusted for children’s age and sex. Cook’s distance tests were used to determine influencing observations on linear regression associations for continuous variables. Further, cross-product interaction term between sleeping time and gender were added to the regression models testing the association between sleeping time and body weight that also included the main effect variables. *P* values <0.05 were considered statistically significant. All analyses were completed with the use of SAS software (version 9.2, 2009, SAS Institute Inc. Cary, NC).

## Results

### Individual and home environment characteristics

A total of 187 Hispanic children participated in the present study (age 11.9 ± 1.4 years, 48% male) and 53% were classified as overweight or obese (Table 1). On average, children reported spending a total of 2.9 ± 2.7 hours per day (h/d) engaged in screen time (ST) behaviours, which is the summation of watching television (TV), playing video games and non-homework computer time. Watching TV accounted for the largest proportion of total screen time (60% of ST). On average, children reported a daily total sleeping time of 8.4 hours; while girls reported slightly higher sleeping time (8.5 vs. 8.4 h/d). Further, younger versus older children (<12 vs. ≥12 years old) also reported higher daily total sleeping time of 9.0 vs. 8.1 hours, respectively. According to the parents, 78% of the children had a screen in their bedroom. About half of the parents (≥48%) limited screen time during weekdays and weekend.

### Sedentary behaviours at home and children’s body weight

Total screen time and the subcategories of time spent playing video game or computer use was not associated with children’s BMI z-score. Total time (h/d) spent watching television (TV) was positively associated with children’s BMI z-score (*P*=0.02). Total physical activity was not associated with children’s BMI z-score. Daily total sleeping time (h/d) was inversely associated with children’s BMI z-score (*P*=0.02); while there was a significant interaction between sleeping time and gender (*P*-interaction=0.02).

We found several independent predictors of TV watching, video gaming and computer use (Table 3). Having a screen in the bedroom was positively associated with children’s total screen time and time spent watching TV (*P*<0.05); while parental limiting was inversely associated with children’s screen time, including total screen time, TV watching and video gaming (*P*<0.05).

## Discussion

The main findings of the present study are the positive associations between time spent TV watching and sleeping with children’s body weight among urban Hispanic children. Having a screening the bedroom was positively associated with children’s total screen time; while parental limiting screen time was inversely associated with these sedentary behaviours. What is very important, however, is that we found no association between physical activity (PA) and BMI z-score; which suggests that total screen time may be an independent predictor of body weight in this population. Further, sleeping time was inversely associated with body weight and this association was modified by gender in that girls reported more sleeping time.

Our main finding extends the findings reported in other ethnic groups [5, 6], to Hispanic youth reported herein [3]. Intriguingly, the data indicates relationship between total screen time and BMI independent of physical activity for Hispanic youth, which had been found in the literature for NHW and African American children [14]. According to the findings in the present study, TV watching displayed the strongest linkage between children’s total daily screen time and children’s body weight, and these findings are in agreement with what others have reported among the youth [7–13]. Surprisingly, despite the prevalence of obesity, the mean daily duration that children reported watching TV was still below the recommended limit of two hours from the American Academy of Paediatrics (AAP). However, total daily screen time, including time spent watching TV, playing video games and using the computer, was higher than the per day recommended by the AAP.

Although, we are still unable to identify a mediator in the association between children’s daily activities and body weight, such as sleep duration or physical activity, there was a positive association between TV time and BMI, and there are multiple theoretical causes that need to be examined more closely. It has been noted in literature that increased sedentary time from sitting in front of a TV might affect energy balance [19]. In theory, this could be a direct effect on physical activity energy expenditure by replacing time spent in physical activity, but this was not supported by our data. Thus, we speculate the effect of total daily screen time either acts through metabolic effects of sedentary behaviour directly or through associated unhealthy eating behaviours. The latter has been speculated to be associated with either exposure to advertising or snacking during the screen time activity.

Two major elements of the home environment have been shown to impact children’s total daily screen time– the presence of a screening the bedroom and parental limits on total daily screen time. The results here suggest that having a screen in the bedroom was not independently correlated with BMI but was correlated to TV watching in accordance with other literature [4, 8, 15, 17]. Yet, contradicting what others have reported, no associations were found with daily video gaming or non-homework computer usage [17]. It may be that our current analysis was underpowered fordetecting this relationship. There is also evidence in the literature to support that parental limits are associated with lower daily television, video gaming, and non-homework computer times [17]. However, parental limits on time spent watching TV was not directly associated with BMI, suggesting a mediating role in the association between times spent watching TV and children’s body weight. The lack of correlation between limits and BMI could have been explained by an indirect relationship through individual screen time activities with parental limits playing a mediating role. There is also the possibility of an reverse relationship – i.e., children who self-selected short screen time and thus did not exceed recommended levels of screen time were not limited by their parents; while, the children who were limited, tended to engage in screen time activities for longer periods of time than those who were not limited.

Future research into this topic should accommodate the limitations of this study. Our study was limited by the modest sample size, short time period and use of self-report diaries. Direct observation or improved monitoring methods could reduce or eliminate possible bias of self-reported data. Finally, a longitudinal study would better account for the long-term effects of screen time and environmental factors on the body weight of Hispanic children.

In conclusion, children’s total daily screen time seemed to contribute to the prevalence of obesity among Hispanic children. Our findings suggest that the home environment plays an important role in Hispanic children’s exposure to TV, video game consoles, and computers and their time spent engaged in these sedentary activities. Although, we did not find an interaction between screen and sleeping time, our findings suggest that sleep time may contribute to higher body weight, although the interaction terms indicated the relationship was complex. Although our study cannot show causality, it is reasonable for family obesity prevention or treatment interventions to include recommendations for parents to remove televisions from children’s bedrooms and monitor total daily screen and sleeping time while increasing physical activity at home as part of daily healthier behaviours. These recommendations may have a positive impact on Hispanic children’s daily life activities and possibly help to reduce their overall risk for obesity in this highly-at risk population.

## Figures and Tables

**Table 1:** Individual and home environment characteristics of 187 middle school urban Hispanic children

Characteristic	Percentage (%) or mean ± SD
**Individual characteristics**	
Age, *years*	11.9 ± 1.4
Gender, *boys*	89 (48)
BMI z-score	0.95 ± 0.95
**BMI percentile categories**	
Healthy weight: ≥ 5% to 85%	47
Overweight: ≥ 85% to < 95%	25
Obese: ≥ 95%	28
**Children’s reported activities, *hours per day***	
Total screen time^a^, (*n*=154)	2.9 ± 2.7
Television (TV) watching, (*n*=177)	1.8 ± 1.8
Video gaming, (*n*=156)	0.6 ± 1.0
Non-homework computer time, (*n*=165)	0.6 ± 1.0
Total Sedentary Time^b^, (*n*=150)	5.3 ± 3.7
Motorized travel, (*n*=181)	1.3 ± 1.2
Phone use, (*n*=178)	1.1 ± 1.8
Total physical activity^c^, (*n*=176)	1.6 ± 1.8
Sports, (*n*=179)	0.7 ± 0.9
Playing, (*n*=179)	0.6 ± 0.8
Non-motorized activities, (*n*=178)	0.3 ± 0.7
Total sleep, (*n*=177)	8.4 ± 1.1
Gender	
Boys, n=89	8.5 ± 1.2
Girls, n=98	8.4 ± 1.0
Age categories	
<12 years old, n=76	9.0 ± 1.1
≥12 years old, n=110	8.1 ± 1.0
**Home Environment characteristics**	
TV, video-game console or computer in the bedroom	78
**Parental limits on TV watching**	
Weekdays	54
Weekends	68
Both	48
**Parental limits on video game time**	
Weekdays	57
Weekends	73
Both	52
**Parental limits on non-homework computer usage**	
Weekdays	59
Weekends	71
Both	54

a Corresponds to the sum of reported time spent watching TV, playing video games, and non-homework computer use

b Corresponds to the sum of reported time in motorized travel, phone use and total screen time

c corresponds to the sum of time play sports, free active play, and non-motorized transport (include walking)

**Table 2:** Associations between children’s reported activities and BMI z-score in 187 middle school urban Hispanic children^a^

Independent variables:	Dependent variable BMI z-score	P value for main effect	P value for interaction sleep*gender^b^
	β(95% CI)		
Total screen time (h/d)	0.04 (−0.01, 0.03)	0.1	
TV watching (h/d)	0.09 (0.01, 0.17)	0.02	
Video gaming (h/d)	0.04 (−0.11, 0.20)	0.5	
Non-HW computer time (h/d)	0.06 (−0.13, 0.26)	0.5	
Total Physical activity (h/d)	−0.01 (−0.09, 0.11)	0.8	
Total sedentary time	0.02 (−0.02, 0.06)	0.3	
Total sleep (h/d)	−0.46 (0.21)	0.02	0.02

Defined: h, hours; d, day

a Values are beta coefficients (β) and 95% confident intervals (CI) for children’s BMI z-score for one unit increase in independent variables except for total sleep values are beta coefficients and standard error. Regression models were adjusted for age and gender.

b Cross-product interaction term between sleeping and gender was added to the regression model for total sleep (h/d) that also included the main effect variables.

**Table 3:** Associations between having a TV, video-games console and/or computer available in bedroom and parental limit on screen time and children’s time spent watching TV, playing video games and using the computer

Independent Variables^a^	Dependent variables	β (95% CI)^b^	P value
Screen(s) in the bedroom Parental limits	Total screen time (h/d)	1.09 (0.25, 1.92) 1.45 (0.59, 2.31)	0.01 0.001
Screen(s) in the bedroom Parental limits	TV watching (h/d)	0.68 (0.22 to 1.14) 0.50 (01 to 1.00)	0.004 0.04
Screen(s) in the bedroom Parental limits	Video gaming (h/d)	0.10 (−0.20 to 0.40) 0.32 (0.05 to 0.60)	0.5 0.02
Screen(s) in the bedroom Parental limits on TV	Non-homework computer use (h/d)	0.06 (−0.18 to 0.31) 0.20 (−0.04 to 0.43)	0.5 0.09

Defined: h, hours; d, day

a Independent variables are categorical binary variables.

b Values are beta coefficients (β) and 95% confident intervals (CI) for children’s BMI z-score for an affirmative answer in independent variables. Regression models were adjusted for age and gender.

## Summary

The prevalence of childhood obesity has increased about 4-fold in the 40 years and only recently has the rate of increase dramatically slowed. The prevalence is now about 17% with higher rates among Hispanics and other minorities. With the higher rates, children are now developing what were previously thought to be adult diseases such as diabetes, high blood lipids, type II diabetes and fatty liver disease. A sedentary lifestyle appears to be major contributor to these diseases and thus the aim of this study was to investigate the associations between the home environment, parental limiting, and screen time with the body weight of Hispanic children from an urban community in the U.S. A total of 187, ages 10–14 years, from a charter middle school in a Hispanic community were enrolled in the study. Questionnaires were completed by the children and their parents.

Although we found that children with the longest time spent in sedentary activities and least sleep tended to have the most excess weight, the relationship between total sedentary time and excess weight was not significant. Time spent watching TV, however, was associated with increased BMI, while sleep time was inversely associated with BMI. These findings suggest that the effects of sedentary activities on BMI in children may not be simply due to periods of low energy expenditure resulting from being sedentary, but that they may involve some other mechanism influencing energy balance.
